# Top-Down Production of Nano-Seeds from Activated Fly Ash Tuned for Enhancing the Early Strength in Blended Cements

**DOI:** 10.3390/nano12142347

**Published:** 2022-07-09

**Authors:** Konstantin Sobolev, Rani Pradoto, Ismael Flores-Vivian, Marina Kozhukhova, Irina Zhernovskaya

**Affiliations:** 1 Department of Civil & Environmental Engineering, University of Wisconsin-Milwaukee, Milwaukee, WI 53211, USA; mik@uwm.edu; 2 Laboratoire de Mécanique et Génie Civil, Université de Montpellier, 34090 Montpellier, France; 3 Construction and Engineering Management Research Group, Faculty of Civil & Environmental Engineering, Bandung Institute of Technology, Bandung 40132, Indonesia; ranipradoto@itb.ac.id; 4 Facultad de Ingeniería Civil, Universidad Autónoma de Nuevo León, San Nicolás de los Garza C.P. 66455, Mexico; ismael.floresvvn@uanl.edu.mx; 5 Department of Materials Science and Materials Technology, Belgorod State Technological University Named after Shoukhov, Belgorod 308012, Russia; ziv_1111@mail.ru

**Keywords:** blended cement, activation, fly ash, vibro-mill, top-down, nano-seed, nanosilica, hydration, strength, structure, cementitious, matrix

## Abstract

To achieve the new level of blended cement performance, the slurries of Class C and F fly ash were mechano-chemically activated in a vibro-mill with superplasticizer and nanosilica. The resulting activated products were tested in mortars replacing up to 30% portland cement. The activation process resulted in the formation of nano-seed clusters and micronized ash particles that both significantly improve the early strength of mortars as well as allow for the replacement of portland cement with industrial by-products. A small amount, 0.1% (of a binder weight), of nanosilica was used in selected compositions to improve the process of activation and facilitate the formation of nano-seeds. Due to an intensive activation of fly ash in the vibro-mill and the formation of nano-seed hydration products, the increase in the heat of the hydration flux and improvement of the mechanical properties such as compressive strength, especially in the early stages of hardening, were achieved. It is envisioned that fly ash activation and the use of supplementary cementitious materials as a precursor can induce a denser structure of cementitious matrix due to better particle packing realized with the application of the nano-seed product, nanosilica, ultra-fine particles of fly ash, and the formation of a refined C-S-H structure realized with the incorporation of the nano-seed particles.

## 1. Introduction

**Figure 1:**
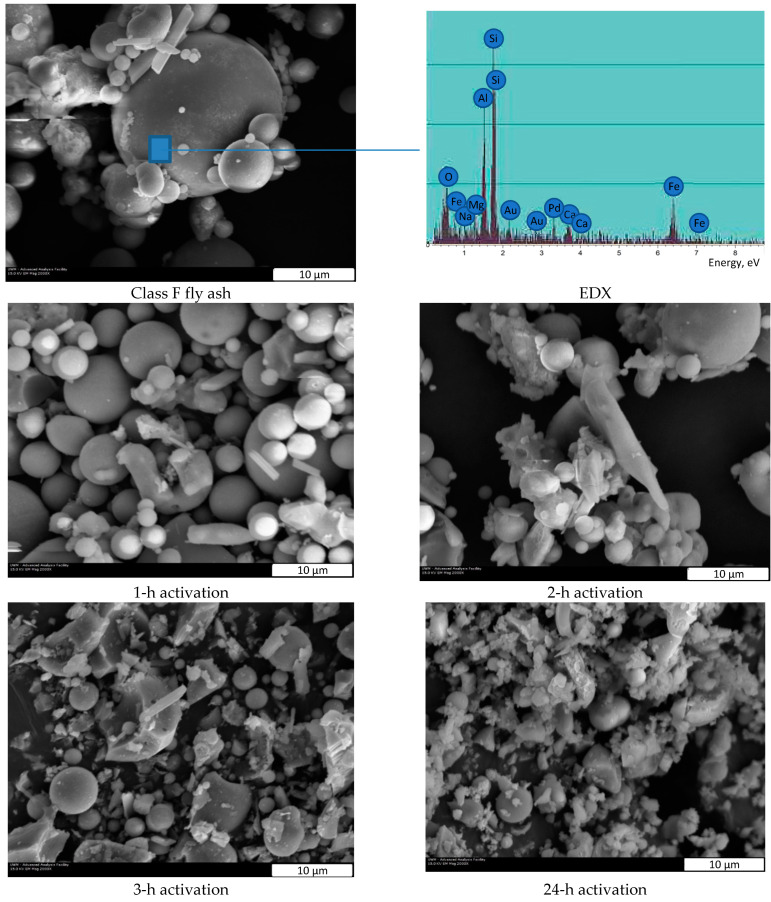
Transformation of Class F fly ash at different activation stages (SEM images at 2000× magnification).

The primary trend in sustainable construction embraces the effective use of concrete with supplementary cementitious materials (SCM) and mineral admixtures, including high-performance, self-consolidating, and other types of high-end concrete [1,2]. In addition to performance enhancement, the use of SCM, which are often large-volume streams of industrial by-products, such as silica fume, fly ash, and ground granulated blast furnace slag (known in the US as slag cement), can reduce the consumption of portland cement by up to 70% [1,2,3,4]. Overall, portland cement compositions with supplementary cementitious and pozzolanic materials such as fly ash (Figure 1) demonstrate excellent long-term cementitious properties comparable to the performance of Roman concrete [3,4,5,6,7,8]. However, because of the hydration delay and “dilution effect”, the incorporation of fly ash into blended cement systems can delay the development of early strength, and this deficiency can be corrected with the use of nanoparticles [5,8,9,10,11,12,13,14,15,16,17,18,19].

The use of nanoparticles in modern concrete comprises another important trend [5,10,11,12,13,14,15]. Submicron and nanosized particles of silica were used to increase the strength and durability of concrete [5,6,7,10,11,12,13,14,15]. It was explained that the addition of nanosilica, when combined with high volumes of fly ash, led to the refinement of pores and decreased the porosity of the cement matrix at the very early ages of hydration [8]. This observation was further explored by Kawashima et al., who studied the effect of nanoparticles such as nano-SiO_2_ and nano-CaCO_3_ on the strength development of fly ash and portland cement-based mortars [19]. Despite the attractive performance, the application of nanoparticles in concrete is still limited, often because of the relatively high costs associated with the use of nanocomponents.

In addition to nanosilica and chemical activation, overcoming poor early-age strength performance through the mechanical activation of cementitious materials has also been proposed [2,5,7]. The mechanical activation of SCM has been realized using a grinding process [20,21,22]; due to the activation and dispersion processes, fly ash products with high fineness can be achieved [2]. Several studies proved that the activation of fly ash through grinding results in higher surface area and, consequently, in higher chemical reactivity [2,3,20,21]. Hela et al. proposed several methods of mechanical activation [21], including the milling of fly ash to improve the rheological properties of blended systems. Smaller reactive particles can act as fillers, thus resulting in a denser structure, higher strength, and improved durability [22]. Therefore, the strength improvement of cement composites can be achieved by the dry milling of individual cementitious components such as fly ash [2,3,22,23,24]. The activated fly ash with enhanced fineness demonstrates higher pozzolanic reactivity and enhanced early strength.

**Figure 2:**
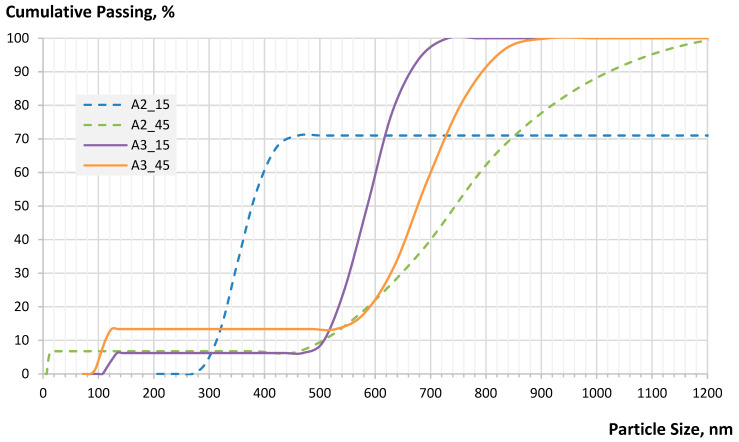
Particle size distribution and zeta potential characteristics of submicron-size portion of activated Class C fly ash after 2 (A2) and 3 (A3) hours of vibro-milling.

A new concept of nano-engineered cement combined with mechano-chemical activation (MCA), accelerated hydration, and the formation of nano-cement and nano C-S-H seed clusters has been previously proposed by the authors [7]. It was concluded that cementitious components subjected to MCA and ultra-fine wet and dry milling could significantly improve strength at all ages of hardening [23]. It is hypothesized that by incorporating nanosilica with high reaction activity and a larger specific surface area, along with fly ash precursor during the wet-milling process, the pozzolanic activity can be significantly increased, as nanosilica accelerates the hydration and promotes pozzolanic reactions. Prior work by the authors has demonstrated the feasibility of producing nano-engineered cement (NEC) with mechano-chemically activated cementitious components through liquid-state milling [7,23]. The activated cement with nano-seeds was used at up to 30% cement replacing levels. The research results demonstrate that the cement hydration and mechanical performance were facilitated by the intensive activation of cement and the formation of nano-seeds, as demonstrated by Figure 2 [7,23]. Fly ash mainly consists of amorphous material [25], and thus the activation carried out by mechanical milling, including wet milling, can improve the reactivity by increasing the surface area available for pozzolanic reactions to occur (Figure 1). In this reported work, wet milling and activation in a vibration mill used as a reactor were employed to reduce the particle size and to boost the reactivity, as well as to trigger the formation of nano-seed products from low-cost fly ash precursor as previously reported [23].

The overall objective of the reported research was to evaluate the feasibility of a new eco-cement concept based on the mechano-chemical activation of fly ash-nanoparticle–superplasticizer blends using liquid-state milling and to illustrate how these processes can be tuned to facilitate the hydration, improve the early strength, and, overall, the performance of blended cement composites.

## 2. Experimental

### 2.1. Materials and Precursors

The portland cement (PC) type I used in this study was supplied by Lafarge-Holcim. The chemical and physical properties of portland cement (as tested by the manufacturer) are given in Table 1, while Table 2 provides the chemical and physical properties of Class C and Class F fly ash used in the experimental program as low-cost precursors for the top-down manufacturing of nano-seeds. 

The mineral composition of fly ash and the effects of activation were determined by X-ray diffraction (XRD) using the XDS 2000 instrument from Scintag (now Thermo Electron Corporation, Waltham, Massachusetts, USA), running on step-scanning mode, and reported in 2Θ units. The particle size distribution and zeta potential of activated ash materials were tested using the Dynamic Light Scattering (DLS) method with a NanoBrook ZetaPALS from Brookhaven Instruments (Holtsville, New York, USA).

Two different types of colloidal nanosilica, supplied by Nouryon (former EKA Chemicals), were evaluated for this study. The Cembinder 50 (CB50) product is characterized by an average particle size of 5 nm and a solid concentration of 15%; Cembinder 8 (CB8) is represented by products with a larger particle size range of 10–100 nm and a solid concentration of 50%, as reported by Table 3. At a dosage of up to 0.5%, nanosilica acts as an effective viscosity-modifying agent [10]. Although the CB8 product is commonly used in concrete, the CB50 material demonstrated better performance in the initial evaluation and was consequently used in the experimental program. 

A polycarboxylate ether (PCE) superplasticizer (SP) with 38.7% solid concentration supplied by Handy Chemicals was used as a surfactant. Graded Ottawa sand complying to ASTM C778 [27] was used as fine aggregate in the mortars. Room-temperature tap water was utilized for the preparation of the activated ash and mortar specimens.

**Table 2:** Chemical and physical properties of fly ash.

Compounds	Chemical Composition, % by Mass			
	Class F	Class C	ASTM C 618 [28] Limits	
			Class F	Class C
SiO_2_	46.9	32.7	-	-
Al_2_O_3_	22.9	17.6	-	-
Fe_2_O_3_	19.2	5.9	-	-
Total, SiO_2_ + Al_3_ + Fe_2_O_3_	89.0	56.2	>70	>50
SO_3_	0.3	2.0	<5.0	<5.0
CaO	3.8	27.3	-	-
MgO	0.8	6.6	-	-
K_2_O	1.7	0.4	-	-
Na_2_O	0.6	2.2	-	-
Moisture Content	0.1	0.8	<3.0	<3.0
Loss on Ignition (Unburnt Carbon)	2.3	0.3	<6.0	<6.0
Physical Properties	Class F	Class C	ASTM C 618 [28] Limits	
			Class F	Class C
Specific Gravity	2.50	2.83	-	-
7-day Strength Activity Index, % of control	77.5	82.9	>75	>75
Water Requirement, % of control	102	91	<105	<105

**Table 3:** Properties of colloidal nanosilica.

Characteristics	Cembinder Type	
	CB50	CB8
Density, kg/cm^3^	1.1	1.4
SiO_2_, %	15	50
pH	10.0	9.5
Viscosity, mPas	<10	<10
Concentration, %	15	51.5
BET Surface Area, m^2^/g	179.4	61.2

### 2.2. Methods Used for Mechano-Chemical Activation and Nano-Seed Preparation

Mechanical activation is an effective method for improving the reactivity of blended cement, as the fine grinding of cementitious materials creates a greater surface area available for hydration. Preliminary research indicated the potential of MCA to significantly improve mechanical properties and to accelerate cement hydration due to the presence of nanosized and submicron phases [7,23,24,25]. In this reported research, the activated component (AC) was prepared using fly ash as a precursor rather than portland cement. A blend of plain ash and AC was used as a cement replacement material at up to 30% by mass. In this study, 500 mL high density polyethylene containers with ash slurry and grinding media were placed on the platform of the vibro-mill and milled for varying activation times (Figure 1). After grinding, the AC slurry was immediately removed from the chamber and transferred to the blending vessel for the preparation of mortar by subsequent mixing with all remaining materials (cement, fly ash, sand, as well as additional nano- and chemical admixtures, as specified in Table 4).

### 2.3. Characterization of Activated Fly Ash Product

To identify the effect of mechano-activation, the Class C fly ash product used in this study was subjected to wet vibro-milling for 2 and 3 h, designated as A2 and A3 set of samples, respectively. After milling, the evaluation of the particle size distribution and zeta potential of the activated ash product were performed using diluted slurries, with the samples collected from the top 20% portion of a container after 15 and 45 min of gravitational sedimentation (further designated as A*z*_15 and A*z*_45 series of samples, where *z* is the activation time in hours), as described in reference [7]. While this method cannot provide a quantitative evaluation of the entire activated product, mainly because of the relatively small portion of nano- and submicron-sized particles (estimated to be <1% by mass), it can provide a clear estimate about the type and stability of such inclusions in the nanomaterial-containing slurry. 

A Brookhaven Instruments Corporation (BIC) NanoBrook ZetaPALS Particle Sizing Instrument was used to obtain the particle size and evaluate the polydispersity. This instrument uses the principle of dynamic light scattering (DLS). Initially, 2 mL of 18.2 MΩ water is placed into a disposable 10 mm square acrylic cuvette. A drop of activated ash suspension (e.g., A2_15, A2_45, A3_15, and A3_45) was then added into the cuvette for each run, and the sample was gently mixed, resulting in a slight turbidity. The cuvette was then placed in the holder of the instrument for the analysis. All tests were conducted at 25 °C, and 10 runs were used to determine the particle size distribution, mean effective diameter, and polydispersity. The same equipment was used to obtain the z- (ζ-) potential of the suspensions. For this experiment, a small amount of activated ash suspension was dispersed in 0.1 mM KNO_3_, resulting in a sample with a slight turbidity. Afterwards, 1.5 mL of sample was then placed into a disposable 10 mm square acrylic cell and the test electrode was inserted. The electrode was then connected to the lead and the cuvette was placed in the holder. Since aqueous media was used to disperse the samples, the Smoluchowski method was used for the calculation. All samples were run at 23 °C and five runs of seven cycles were used to obtain the mean value for the z-potential.

The results of the DLS and zeta potential tests are depicted in Figure 2. After 2 h of activation, followed by 45 min (A2_45) of gravitational sedimentation, the sample was represented by up to 7% nanosized fraction of fly ash with the size of 6–14 nm. The zeta potential value for this slurry was also the highest, demonstrating better electrochemical stability vs. the other activated products. The slurry activated for 3 h subjected for 45 min of the sedimentation (A3_45) also demonstrated the presence of a nanosized fraction, which is shifted towards the larger particle size range of 90–100 nm. The zeta potential for this slurry was higher in comparison with the values obtained for the slurries A2_15 and A3_15. The increase of the average particle size of the slurry as the vibro-milling time increases can be explained by high chemical activity and so the tendency to agglomeration of the Class C fly ash product. 

This experiment proves that after 2 h of wet vibro-milling, some portion (up to 7%) of colloidal fly ash particles can be reduced to nano size (<100 nm).

### 2.4. Design of Experiment

The mortars based on blended binders with activated ash were produced at a water to cementitious materials ratio (W/C) of 0.36 and a sand to cementitious materials ratio (S/C) of 1, as further presented in Table 4.

Two experimental approaches were explored to realize the proposed concept: first, mechano-chemical activation was used to activate Class F fly ash, which was used at a replacement level of 20%, and combined with an additional 10% of non-activated (plain) fly ash. Here, the activation of 20% of Class F fly ash was carried in a water solution with superplasticizer (SP), and the remaining 10% of the dry fly ash was added with the cement at the mortar-mixing stage. 

The SP was used at a constant dosage of 0.15% by mass of cementitious materials to facilitate the dispersion of fly ash during the liquid-state milling (when used in activated systems). The fly ash component was activated in a vibro-mill at a grinding media to powder ratio of 10:1, leading to an effective reduction of particle size and a change in particle shape, as demonstrated in Figure 1. The effects of milling times of 1, 2, 3, 4, and up to 24 h were investigated. All experimental results for the activated compositions were compared with the performance of superplasticized mortars (R30) containing 30% plain fly ash (no activation). Therefore, all mortars had an identical composition, but utilized different production routes. The objective of this research phase was to determine the optimal activation time for improved mechanical performance of blended systems. 

The second phase of the research was conducted to compare the effects of activated Class C and Class F fly ash used at a 20% replacement level to the performance of portland cement mortar (R). This experiment used fly ash obtained with an optimal activation time of 3 h and included the testing of mortars at W/C of 0.3 and S/C of 1. To produce the AC, the required quantities of fly ash (20% of the total mass of cementitious materials) were pre-mixed with tap water, superplasticizer, and, optionally, nanosilica. The resulting fly ash blend was activated by vibro-milling and used for the preparation of mortars as discussed for the first part of the experiment. 

### 2.5. Methods of Mortar Specimen Preparation and Testing

The preparation of the mortar specimens was based on ASTM C109 [29] and C305 [30], updated for chemical admixture and nanoparticles testing [7]. The workability and density of fresh mortar were evaluated as specified by ASTM C1437 [31] and C138 [32]. Mortar flow was tested using a 254 mm flow table using ASTM C230 standards [33]. Isothermal conduction calorimetry was used to evaluate the hydration kinetics and setting times using the thermal power “heat flow” at different times using the ASTM C1702 method [9,22,23,24,34,35]. The TAM Air unit from TA Instruments, which demonstrated excellent performance in the evaluation of portland cement interactions with chemical admixtures and supplementary cementitious materials, was used in the experimental program [34]. In this reported experiment, the same mortars as those used for the evaluation of fresh and hardened properties were tested for heat flow. 

To examine compressive strength, cube mortar specimens with side dimensions of 50.8 mm were cast and cured in accordance with ASTM C109 [31]. Test specimens were removed from the molds after 24 h of curing, immersed in lime water, cured at a temperature of 23 ± 2 °C, and then tested at the age of 3, 7, and 28 days. The compressive strength of each mortar specimen was determined using a standard loading rate of 1.4 kN/s. The reported values represent an average of at least two specimens tested for each age.

## 3. Experimental Results and Discussion

### 3.1. Transformations of Fly Ash Induced by Mechano-Chemical Activation

The microstructure of activated fly ash was investigated to quantify the process of mechano-chemical activation. Activated Class F fly ash was characterized using X-ray diffraction (XRD) analysis and scanning electron microscopy (SEM) for specimens activated for 1, 2, 3, and 24 h (Figure 1). Superplasticizer and nanosilica were added (as specified by experimental matrix) to facilitate liquid-state dispersion during milling. Longer processing of up to 24 h led to the formation of smaller particles (less than 3 μm), but 3-hour treatment proved to be sufficient to reduce the particle size to less than 10 μm.

**Figure 3:**
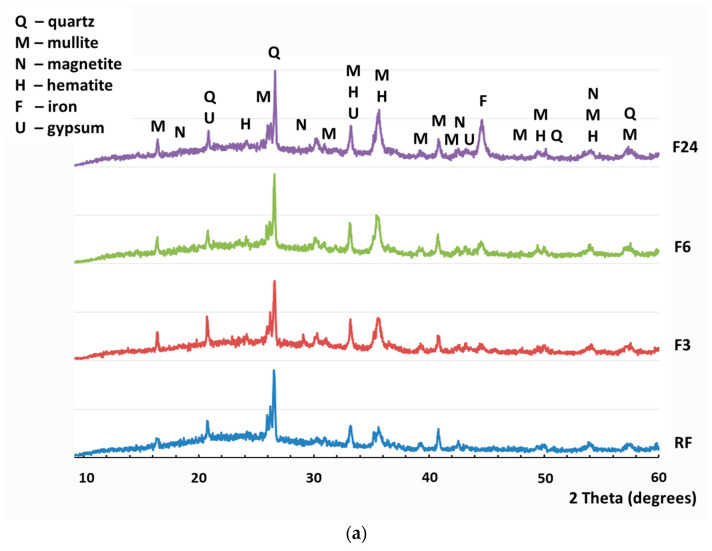

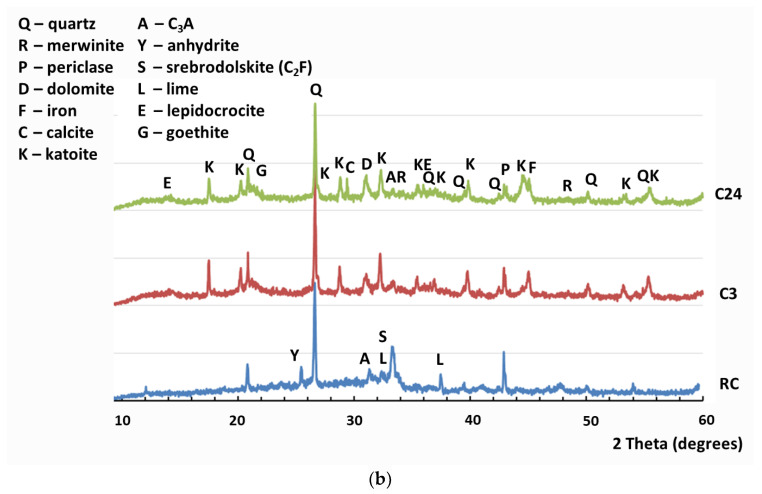
Transformation of (**a**) Class F fly ash (RF is a reference Class F fly ash; F3, F6, and F24 are Class F–based products activated for 3, 6, and 24 h, respectively) and (**b**) Class C fly ash (RC is a reference Class C fly ash; C3 and C24 are Class C products activated for 3 and 24 h, respectively) due to wet vibro-milling up to 24 h.

The results of the XRD analysis for reference Class F fly ash and specimens activated for 3, 6, and 24 h (RF, F3, F6, and F24, respectively) are depicted in Figure 3a. The intense quartz and mullite peaks are consistent with the high silica content expected in such samples, while the presence of other minerals such as anhydrite gypsum, magnetite, and hematite were found in small quantities (<10%) in the crystalline phase of the Class F fly ash. Further Rietveld refinement indicated that the bulk of the mineral composition, about 70%, was represented by the amorphous glass phase, a clear sign of the high pozzolanic reactivity of the ash. 

The XRD characterization carried out after the activation process returned an increased intensity of magnetite peaks observed at 30.15° (220); 35.52° (131); 43.17° (400); 57.12° (115), and reflections of iron at 44.55° (110) were observed (comprising up to 10% by weight of the crystalline phase in C24 samples corresponding to 24 h milling), as reported in Figure 3a. The increased proportion of magnetite corresponds to iron release from the milling yield, which increased up to 3% and 8.5% upon milling for 3 and 24 h, respectively. Partial oxidation of iron to magnetite can occur with milling, although the increase of hematite at 24.16° (012); 33.18° (104); 35.68° (112); 49.53° (024) portion in the composition was not significant. Here, the use of stainless-steel grinding media for the activation of Class F fly ash generates a significant amount of milling yield that is partially subjected to oxidation to magnetite (Fe_3_O_4_) and leads to a considerable concentration of this phase in the activated composition. In the case of Class F ash, there is no evidence of the formation of new nanosized products capable of accelerating the hydration of portland cement systems. The use of inert grinding media such as corundum can be recommended to avoid the contamination and potential reactivity reduction of Class F fly ash.

Based on XRD, the main cementitious component of Class C fly ash capable of hydration and the formation of nano-seeds is C_3_A, which is found in a crystalline phase at 32%, Figure 3b. Upon 3-hour hydration in the mill, the formation of a new phase of katoite (Ca_3_Al_2_(SiO_4_)_3-*x*_(OH)_4*x*_, where *x* = 1.5–3) was detected, reducing the C_3_A content to 11%. It can be observed that the C_3_A phase was completely converted to katoite after 24 h of milling. However, the follow-up activation for 24 h resulted in the contamination of a specimen with iron at the level of up to 12%. Furthermore, extended milling results in the formation of considerable volumes of iron hydroxide phases such as lepidocrocite γ-FeO(OH) and goethite α-FeO(OH), the contribution of which to the hydration of portland cement systems is not clear. Therefore, it can be concluded that wet mechanical activation in a mill for up to 3 h results in the formation of potentially beneficial nano-seed phases derived from C_3_A available in Class C fly ash, and the overall process can be described as mechano-chemical activation.

### 3.2. Hydration Process

**Figure 4:**
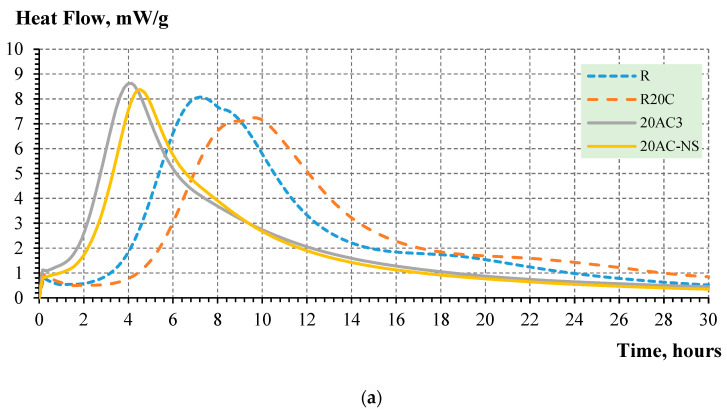

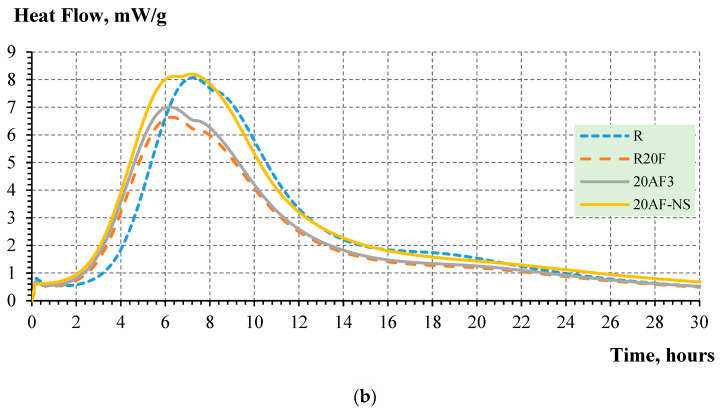
Effect of activated fly ash on the hydration heat evolution: (**a**) Class C fly ash; (**b**) Class F fly ash.

Based on the heat of the hydration experiment, it was observed that the activation of fly ash in a vibro-mill considerably accelerates the hydration of cementitious systems with fly ash vs. reference mix (Figure 4). The best performance was reached by the composition with activated Class F fly ash and nanosilica. Here, the formation of nano-seed products was not detected; thus, this effect is achieved mainly due to the contribution of ultrafine fly ash particles and nanosilica.

It appears that 3-hour activation results in the considerable performance enhancement of blended cement systems. Specifically, the application of activated fly ash enables the acceleration of hydration of portland cement-fly ash blends vs. reference portland cement mortar (R30C), as demonstrated in Figure 4b. Furthermore, the mechano-chemical activation leads to the reduction of the dormant period and setting times, and can potentially enhance the early strength of blended cementitious composites with up to 20% of Class C fly ash. In the systems with Class C fly ash, the role of nanosilica was less pronounced due to the formation and predominant effect of nano-seeds from activated ash [7,22].

### 3.3. Properties of Mortars

While all of the mortars were produced at the same W/C ratio of 0.3, the workability of the mortars with activated fly ash was improved with an increase in the activation time of up to 4 h. This is achieved due to a better packing of the particulate systems with activated fly ash and can be used to design concrete mixtures of the same workability at a reduced W/C vs. reference [20,21,25,26]. The compositions with nanosilica had an enhanced segregation resistance, but at a cost of reduced workability, which was still better than that observed for the reference mortar (R) [19]. This system with activated ash and nanosilica can be beneficial for the design of self-consolidating concrete [10,17,18]. It can be observed that in addition to the improvement of the rheological properties and segregation resistance of activated slurries, nanosilica can be potentially used to boost the early strength performance of binders with fly ash, as further demonstrated by the results of the reported investigation. 

**Figure 5:**
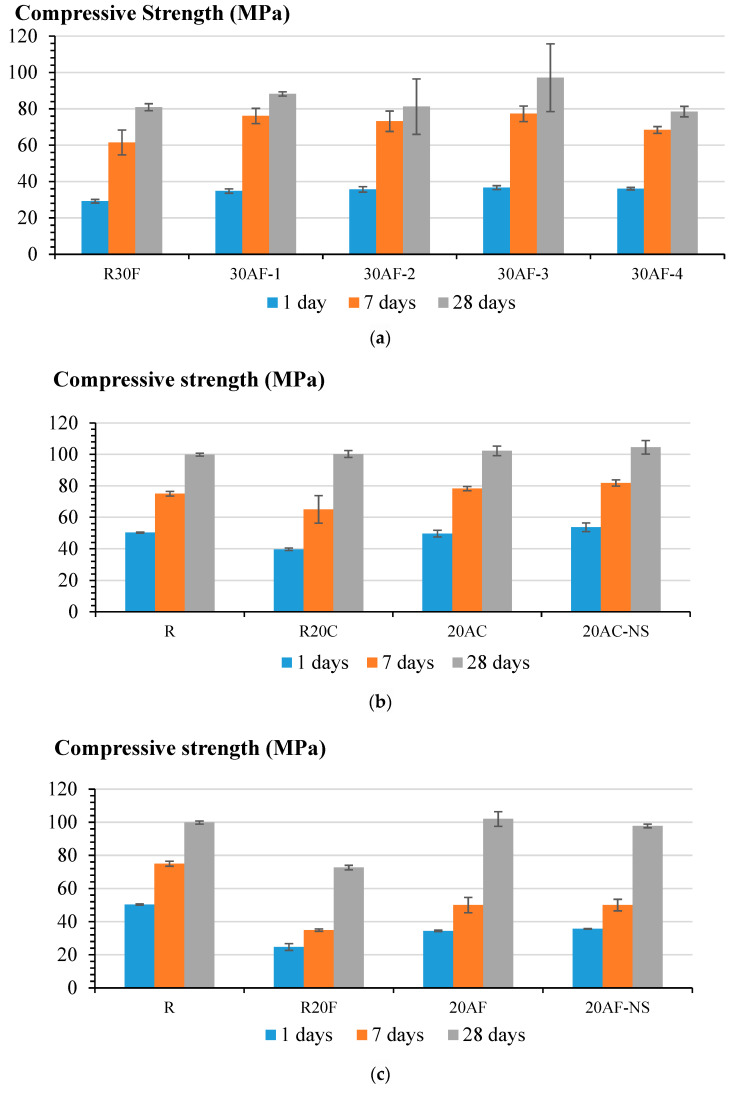
Effect of activated fly ash and nanosilica on the compressive strength of mortars: (**a**) the effect of activation time; (**b**) Class C fly ash; (**c**) Class F fly ash.

Figure 5a demonstrates that the use of 20% of activated Class F fly ash in combination with 10% of plain (non-activated) fly ash improves the strength of blended cement mortars at all ages of hardening. Specifically, the 1-day and 7-day compressive strength of mortars with activated Class F fly ash was considerably improved. The best results were achieved in the systems with fly ash activated for 3 h (Figure 5a). 

Likewise, the incorporation of 20% of activated Class C fly ash results in the improvement of the 28-day compressive strength, which was comparable to the performance of the reference portland cement. The main effect of activation is in the improvement of 7-day strength (Figure 5b) due to the presence of ultrafine particles and the nano-seed effect observed for the activated Class C fly ash. Overall, mortars based on activated Class C fly ash had better performance than the reference portland cement at all ages of hardening. This is achieved due to the ultrafine milling and activation of the fly ash. While systems with 20% of activated Class F fly ash had 28-day compressive strength comparable to the reference portland cement mortar, the 1-day and 7-day strength of this system was about 31% and 33% lower, respectively, vs. the reference (R) (Figure 5c).

## 4. Conclusions

This paper describes a series of experiments evaluating the impacts of mechano-chemically activated fly ash precursors on the rate of hydration, the structure of the hydrated products, and the mechanical properties of mortars.

It was proved that the use of mechano-chemical activation of a Class C fly ash precursor with superplasticizer and, optionally, with nanosilica, enables the top-down formation of an activated nano-seed product capable of considerable enhancement of the strength performance of blended cement-based composites. The X-ray diffraction and scanning electron microscopy investigations prove that the activated ash material consists of finely ground and, in the case of Class C fly ash, hydrated nanosized products that are stabilized in a slurry containing chemical admixtures.

With isothermal calorimetry, it was demonstrated that the use of activated ash can increase the rate of hydration and, hence, can boost the development of early-age strength. It was found that with activated ash replacement, the 1-day compressive strength of blended binders is increased by up to 26% (for Class F fly ash). Here, the improved packing density from milled particles of fly ash produces a denser composite matrix, which enhances the overall strength development [1,2,3,4,5,6,7]. The increase in 28-day strength of developed blended composites with 20% cement replacement by Class C and Class F fly ash was comparable to the reference portland cement mortars.

The activated Class C fly ash was overperforming Class F fly ash in the blended cement systems due to its cementitious properties and also the presence of ultrafine activated ash and nano-seeds. Still, the activation was very effective in Class F fly ash systems, considerably enhancing the compressive strength at the age of 28 days; it can be concluded that for Class F ash, this effect is determined by particle size reduction and improved surface reactivity. Therefore, the boost of the performance of cementitious systems with activated fly ash is due to the presence of ultrafine super-reactive particles of fly ash, nanosilica, and, in the case of Class C fly ash, nano-seed product represented by katoite, with all of these features resulting in the enhancement of performance.

Further research is necessary to identify the effects of nano-seeds from an activated fly ash precursor on the composition and morphology of the hardened cement matrix, including the mature systems cured over 28 days, the pore size and permeability modification, as well as the long-term durability performance of developed blended cement composites with an activated component.

## Figures and Tables

**Table 1:** Chemical composition and physical properties of Portland cement.

Chemical Composition	ASTM C150 [26] Limit, %	Result, % by Mass	Physical Properties	ASTM C150 [26] Limit	Test Result
SiO_2_	-	19.4	Specific gravity	-	3.08
Al_2_O_3_	-	5.3	Time of setting, minutes		
Fe_2_O_3_	-	3.0	Initial	45 min	88
CaO	-	63.2	Final	375 max	222
MgO	6.0 max	2.9	Compressive strength, MPa		
SO_3_	3.0 max	3.3	1 day	-	18.1
Loss on Ignition	3.0 max	1.1	3 days	12.0	28.7
Na_2_O	-	0.3	7 days	19.0	34.3
K_2_O	-	0.7	28 days	28.0	40.4
Others, %	-	0.9			
C_3_S		60.7			
C_2_S	-	9.9			
C_3_A	-	8.9			
C_4_AF	-	9.1			
C_4_AF + 2(C_3_A)	-	26.9			
C_3_S + 4.75(C_3_A)	-	102.9			
Na_2_O_eq_	0.6 max	0.8			

**Table 4:** Experimental matrix and performance characteristics of mortars.

Mix ID	Cement Replacement with Fly Ash, % by Mass	W/C	Activation Time, Hours	Composition of Activated Component, % by Mass			SP, %	Flow, %	Setting Time, Minutes	
				Fly Ash		Nano Silica			Initial	Final
				Class F	Class C					
R30F	30	0.36	-	-	-	-	0.15	82	210	450
30AF1	30	0.36	1	20	-	-	0.15	83	210	435
30AF2	30	0.36	2	20	-	-	0.15	94	180	372
30AF3	30	0.36	3	20	-	-	0.15	108	165	332
30AF4	30	0.36	4	20	-	-	0.15	>140	150	306
R	-	0.3	-	-	-	-	0.15	47	84	420
R20C	20	0.3	3	-	-	-	0.15	42	228	582
20AC	20	0.3	3	-	20	-	0.15	105	54	240
20AC-NS	20	0.3	3	-	20	0.1	0.15	90	60	258
R20F	20	0.3	3	-	-	-	0.15	>140	108	324
20AF	20	0.3	3	20	-	-	0.15	>140	108	300
20AF-NS	20	0.3	3	20	-	0.1	0.15	130	108	348

## Data Availability

Additional test data can be available on request.
